# An Actively Homing Insertion Element in a Phage Methylase Contains a Hidden HNH Endonuclease

**DOI:** 10.3390/genes16020178

**Published:** 2025-02-01

**Authors:** Danielle Arsenault, Sophia P. Gosselin, Johann Peter Gogarten

**Affiliations:** 1 Department of Molecular and Cell Biology, University of Connecticut, Storrs, CT 06268-3125, USA; danielle.arsenault@uconn.edu (D.A.); sophia.gosselin@uconn.edu (S.P.G.); 2 Institute for Systems Genomics, University of Connecticut, Storrs, CT 06268-3125, USA

**Keywords:** homing, selfish genetic element, actinobacteriophage, endonuclease

## Abstract

**Background/Objectives**: The ShiLan domain was previously identified as an insertion sequence in a phage DNA methylase gene that exhibited similar evolutionary patterns to that of an active intein or self-splicing intron but could not be identified as either. It produces no internal stop codons when read in frame with its host methylase gene, leading to the thought that it may not be an intron and rather be an abnormal type of intein. However, the sequence has no detectable self-splicing domains, which are essential for intein persistence, as preventing an intein from successfully splicing is often detrimental to proper host protein function. **Methods**: The analysis of alternate open reading frames for the full nucleotide sequence of this insertion element revealed the insertion to be an out-of-frame histidine-asparagine-histidine (HNH) endonuclease. A GTG start codon is located 18 bp into the insertion, and a TAA stop codon within the last four bases of the insertion (TAAC). When this frame is read, an HNH endonuclease is revealed. In-depth computational analysis could not retrieve support for this element being any known type of self-splicing element, neither intein nor intron. When read in-frame with the methylase gene, this insertion is predicted to take on a looping structure that may be able to avoid interference with the DNA methylase activity. We performed searches for sequences similar in nature to the inserted out-of-frame HNH and found several in other phages and prokaryotes. We present our survey of these out-of-frame endonuclease insertion elements as well as some speculation on how these endonucleases are getting translated to facilitate their homing activity. **Conclusions**: These findings expand our understanding of the possible arrangements for and prevalence of unorthodox mobile genetic elements and overlapping open reading frames in phages.

## 1. Introduction

### 1.1. Inteins

Inteins, or intervening proteins, are mobile genetic elements that frequently engage in horizontal transfer during the active stage of their life cycle and can be transferred between divergent individuals. As such, inteins can be used to aid the identification of avenues for recombination between organisms or phages that may be missed by the study of traditional genes that are primarily vertically inherited [1]. Inteins invade their host gene in a manner similar to that of self-splicing introns that encode homing endonucleases. The homing endonuclease usually either comprises a domain of the intein or is encoded as an open reading frame (ORF) on the intron. To promote propagation of the homing endonuclease-containing intein or intron sequence, the homing endonuclease enzyme catalyzes a double-strand cut in an intein-free or intron-free homologous gene. During repair of the double-strand cut by the cell’s repair machinery, the invaded gene can be used as a template, and the intein or intron is copied into the previously uninvaded homolog. In contrast to introns, inteins are not post-transcriptionally spliced out at the RNA level. Instead, inteins are translated together with their host protein and engage in post-translational protein splicing. Inteins use their self-splicing domains to excise themselves from their host protein and ligate the two halves of the host protein together forming a peptide bond, yielding the free intein and the fully functional host protein [2,3,4]. The unorthodox evolutionary behavior and unique enzymatic capacity of inteins make them important elements to further characterize, as they are still understudied despite being present across all domains of life [5]. As ever-growing numbers of genome sequences become available to the public, the pool of systems from which inteins can be extracted and explored continues to grow dramatically. Such explorations require expansive yet meticulous computational analyses to lay foundations off of which future wet lab-based experiments can be informed.

### 1.2. SEA-PHAGES and the ShiLan Domain

Our recent work retrieving and characterizing the hundreds of intein sequences present in the massive Actinobacteria phage (Actinobacteriophage) genome database PhagesDB [6] has revealed incredible Actinobacteriophage intein diversity and disjunct distribution of individual inteins [7]. We incorporated a portion of this intein characterization work into the University of Connecticut’s bioinformatics component of its SEA-PHAGES (Science Education Alliance—Phage Hunters Advancing Genomics and Evolutionary Science) course-based undergraduate research experience. In recent iterations of the course, we provided each student with their own unique insertion sequence to characterize that we had previously identified as being a putative intein. Throughout the characterization of their sequence, the students learn and practice many types of bioinformatic analyses and often end up making fascinating observations. In previous work [8], we reported on a cryptic insertion sequence, deemed the ShiLan domain, found in a DNA methylase gene in the phage ShiLan (ShiLan gene product (gp) 65). This sequence was first brought to our attention during a student’s investigation of a separate insertion sequence, which has since been characterized as an intein, also present in the ShiLan gp65 methylase. Meticulous manual inspection and comparison between related methylase sequences revealed this methylase to have three identifiable insertions with varied presence across different phages from clusters A1, E, F1, J, and P (see [9] for a description of the clustering approach): a seemingly lone LAGLIDADG (named after its characteristic LAGLIDADG amino acid motif) homing endonuclease for which neither an intein self-splicing domain nor a supporting self-splicing intron could be computationally detected, the intein, and a third unrecognizable 202 amino acid (aa) long insertion that we called the ShiLan domain after the phage it was first discovered in. The methylases containing the lone LAGLIDADG homing endonuclease group together in phylogenetic reconstruction and thus this insertion appears to be vertically inherited. In contrast, the intein and the ShiLan domain have disjunct distributions that are different from each other [8]. The ShiLan domain was found in methylases from phages classified in PhagesDB as belonging to the E, F1, J, and A1 clusters. The ShiLan domain insertion does not produce any internal stop codons when read in-frame with the methylase gene and inserts between two codons in a handful of highly conserved residues, just as inteins do. Curiously, this sequence read in-frame with its host gene does not encode any of the essential amino acid sequence motifs known to be important for forming intein self-splicing domains [4,10] nor a homing endonuclease domain. We generated a predicted protein structure for this element using AlphaFold [11] and found that it did not resemble any components of an intein and had overall poor confidence, barring a small central helical bundle. Additionally, the sequence cannot be readily classified as any known type of intron based on its nucleotide sequence or predicted RNA secondary structure. In our initial work, we deemed this insertion element the ShiLan domain and presented, to our best knowledge, all we could ascertain about its identity, function, and distribution [8].

### 1.3. The ShiLan Domain Contains an Out-of-Frame HNH Endonuclease

Subsequent revisiting and analysis of the ShiLan domain after the discovery of histidine-asparagine-histidine (HNH) endonuclease-like insertions in other phage methylases during our ongoing mass characterization of the inteins in PhagesDB as of November 2023 [7] included assessing the products of the other five possible reading frames. Incredibly, using the GTG codon 18 base pairs (bp) into the insertion sequence as a start codon, the ShiLan domain encodes a hidden HNH endonuclease in the third frame relative to the frame of the DNA methylase. The last four nucleotides of the insertion sequence are TAAC, encoding the stop codon in the third frame and denoting the end of the HNH endonuclease product (195 aa). The product contains a central HNH domain and flanking zinc finger domains. We present our analysis of this ShiLan domain/out-of-frame HNH endonuclease and highly similar elements and discuss how their protein products may still be getting translated to facilitate the homing capacity that this element demonstrates, as reflected in its disjunct distribution across closely related phage methylases. Through large-scale in-depth computational analyses, we are collectively becoming increasingly aware of the novel and unorthodox types of mobile genetic elements present in phages [7,12], with the ShiLan domain and similar hidden HNH endonucleases joining this ever-growing collection of elements awaiting future exploration and characterization.

## 2. Materials and Methods

### 2.1. Protein Structure Prediction and Comparisons

All protein structures were predicted using AlphaFold v.2.3.2 [11] and analyzed in ChimeraX v.1.8 [13]. The structural alignments were performed using the Matchmaker tool in ChimeraX. The experimentally solved protein structures used for comparison to predict protein structures included the following: full intein PI-SceI (PDB 1LWS [14]), mini intein (only contains a self-splicing domain, no homing endonuclease domain) from a *Mycobacterium* GyrA protein (PDB 1AM2 [15]), a phage HNH endonuclease (PDB 5H0M [16]), HNH homing endonuclease I-HmuI (PDB 1U3E [17]), HNH homing endonuclease I-TevI (PDB 1I3J [18]), and HNH endonuclease PacI (PDB 3LDY [19]). Versions of the predicted structures colored by model confidence values (pLDDT, predicted local distance difference test [11]) are provided.

### 2.2. Databank Searches and Sequence Comparisons

The protein sequence-based basic local alignment search tool BLASTP [20] was used to search against the NCBI non-redundant database using the original frame and HNH frame translations of the ShiLan domain from phage ShiLan. All default settings were used. The predicted secondary structure-based protein structure database search tool Foldseek [21] was used to search multiple protein structure databases using the original frame and HNH frame translations of the ShiLan domain from gp65 of phage ShiLan. All default settings were used. The profile hidden Markov model-based protein database search tool HHpred [22] was used with the original frame and HNH frame ShiLan domain as queries and used to search against the following protein databases: PDB_mmCIF70_16_Aug [23], UniProt-SwissProt-viral70_3_Nov_2021 [24], PHROGs_v4 [25], and Pfam-A_v37 [26]. The web-based tool for searching against NCBI’s conserved domain database [27] was used with the original frame and HNH frame ShiLan domain as query. All default settings were used. Gene maps and alignments were visualized with Geneious [28], Phamerator [29], and SeaView [30] throughout.

### 2.3. RNA Secondary Structure Prediction

RNAFold [31] was used to generate predicted minimum-free-energy RNA secondary structures. These structures were compared to the known variations of self-splicing introns, in particular, group I introns [32], as this would be the most likely identity for these elements out of any known introns.

### 2.4. Intron Detection with RNAweasel Through MFannot

The intron identification program RNAweasel [33], which is designed to be especially effective for detecting group I and group II self-splicing introns, is implemented in the genome annotation program MFannot [34]. MFannot was used to annotate the entire ShiLan DNA methylase sequence in search of any sign of a self-splicing intron and was also run on phage LittleE’s major capsid group I intron for comparison. Geneious [28] was used for gene map visualizations

## 3. Results

### 3.1. Assessing the Similarity of ShiLan Domain Nucleotide Sequences to Self-Splicing Introns

**Figure 1:**
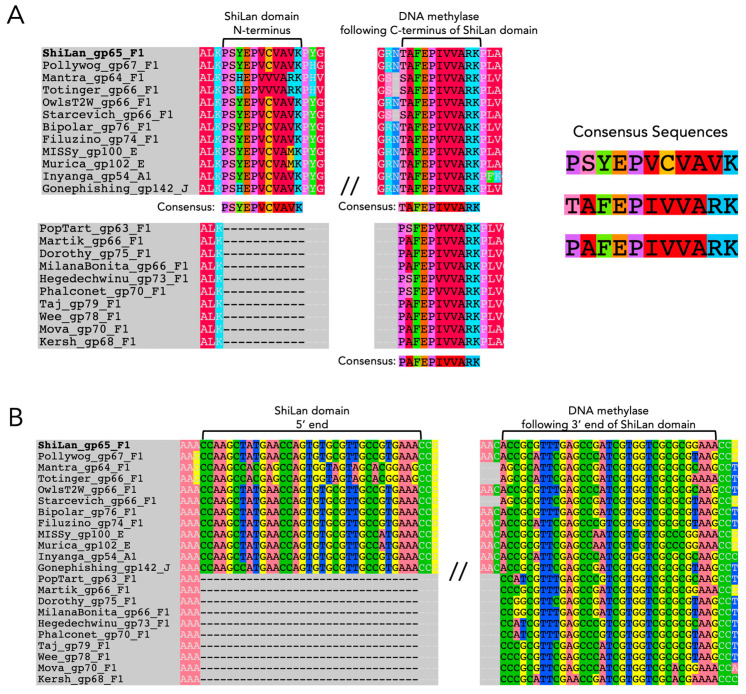
Comparison of the N-terminal/5′ end of the ShiLan domain insertion sequence to the sequence of the methylase on the C-terminal/3′ side of the insertion site. (**A**). Amino acid sequence alignment of homologous DNA methylases from several phages, some containing the ShiLan domain insertion, and some not. The gene product (gp) number and phage cluster to which each phage belongs is denoted next to their name. The start and end of the ShiLan domain sequence are pictured. The first 11 amino acid residues of the ShiLan domain insertion and the first 11 amino acid residues of the methylase on the C-terminal side of where the ShiLan domain inserts itself have darker text and are indicated with brackets. Consensus sequences for the amino acid regions described are provided. Amino acid residues are colored by functional group. (**B**). Nucleotide sequence of the regions described in (**A**).

Comparing the ShiLan domain insertion sequences read in frame with the methylase from phage ShiLan and nine other phages, we found minute similarities to self-splicing regions of group I introns and inteins, though it is not known if these similarities are sufficient for this element to self-splice post-transcriptionally or post-translationally. With respect to introns, the first 11 amino acid residues of the insertion sequence when translated in the frame of the methylase are very similar to the first 11 amino acid residues on the C-terminal side of the insertion site (Figure 1A). This stuck out as a potential self-splicing group I intron signature, as there have been many observations of the 5′ end of a group I intron bearing incredibly high semblance to the 3′ side of its insertion site at the nucleotide sequence level, possibly due to both of these regions needing to interact with the internal guide sequence of the intron for successful self-splicing [35]. While the invaded methylase sequences showed a lower level of similarity to the N-terminus of the insertion in their C-terminal side of the insertion site than the uninvaded sequences (pairwise percent identities of 69.5% versus 75.9%, respectively), we decided to see if the similarity held at the nucleotide level regardless, as nucleotide sequence similarity would be a more promising signifier of similarity to a self-splicing intron. The similarity is not quite as striking from start to finish overall at the nucleotide level (pairwise percent identities of 65.7% and 67.8%, respectively) (Figure 1B) as it is at the amino acid sequence level, providing less support for this element’s identity as a group I intron. Countering the small amount of support for this insertion being an intron provided by these sequence similarities, the insertion sequences also lack commonly observed motifs of group I or group II self-splicing introns, such as a G at the 3′ end for group I introns, or the sequence GTGCG at the 5′ end for group II introns [36]. Pope et al. identified an unorthodox self-splicing intron in gp34 (minor tail protein) of the phage BAKA, which begins with TTGT and ends with ACCG, which they showed, via RT-PCR, is capable of self-splicing [12]; however, there is no sequence similarity between the 5′ and 3′ ends of this intron and the ShiLan domain sequence. Additionally, MFannot was still able to detect the BAKA minor tail intron as a group I-derived intron (e-value 8.25 × 10^−7^, full output available in the provided GitHub repository [37]), whereas no trace of any intron type was detected for the ShiLan domain.

### 3.2. Assessing the Similarity of ShiLan Domain In-Frame Amino Acid Sequences to Inteins

**Figure 2:**
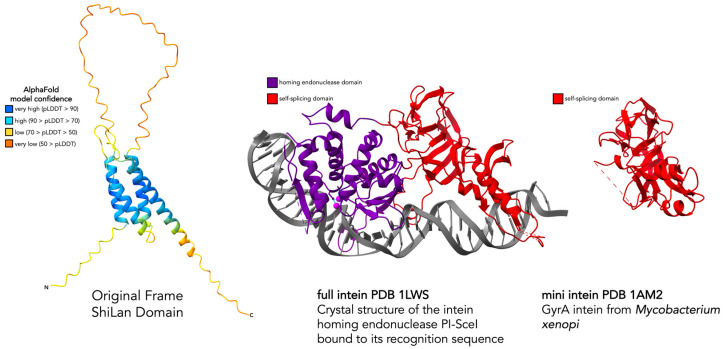
Comparison of the predicted protein structure for the ShiLan domain read in the frame of the methylase to solved structures of full and mini inteins. AlphaFold [11] was used to generate a predicted protein structure for the ShiLan domain read in the frame of the methylase it invades. AlphaFold-provided pLDDT confidence values were used to color the structure (left). Solved crystal structures of a full intein (contains a homing endonuclease and a self-splicing domain) and a mini intein (only contains a self-splicing domain and has lost its homing endonuclease domain), the PDB entries 1LWS [14] and 1AM2 [15] respectively, are provided for comparison (right). The ShiLan domain predicted structure does not resemble either type of intein.

An initial aspect of these insertion sequences that brought us to investigate whether or not they are inteins as opposed to introns was that all of the invaded sequences have a threonine or serine as their first residue on the C-terminal side of the insertion site, whereas the uninvaded sequences have a proline (Figure 1A). For the known intein splicing mechanisms, a threonine (T), serine (S), or cysteine (C) is required as the first residue on the C-terminal side of the intein insertion site [4]. In contrast, having proline as the first residue on the C-terminal side of the insertion site has been shown to inhibit an archaeal helicase intein’s ability to splice, even if recognition and invasion are initially successful [38]. It has been speculated that some inteins bring along a splicing-friendly residue (T, S, or C) with them when invading in the form of a conversion track resulting from the recombination event that allowed them to invade. However, evidence against this element being any known type of intein comes from the two different flavors of C-terminal endings. For the majority, there is an asparagine as the last residue of the in-frame translation of the element, which is another crucial residue for intein splicing [4]. In contrast, for cases such as Mantra and Starcevich, the insertion ends on a serine instead of an asparagine, which has not been observed in any known intein. Two even stronger pieces of evidence against this element being an intein are that, as previously mentioned, the in-frame translation lacks motifs known to be important for intein self-splicing [10], and its predicted protein structure in no way resembles an intein (Figure 2). Throughout this work, we generated several predicted protein structures and provide comparisons to experimentally solved structures where possible. While sequence-based structural predictions are a useful tool for preliminary speculations on potential function, they should be interpreted with caution and nuance. Having experimentally solved structures to corroborate regions of interest on a predicted structure increases the confidence one can have in the accuracy of those predictions.

### 3.3. Comparing Original Frame and HNH Frame ShiLan Domain Protein Products

The effort to ascertain the identity of the ShiLan domain was resumed after finding HNH endonuclease-like insertions in other phage methylases during our recent large-scale characterization of the inteins in PhagesDB [7]. Taking the nucleotide sequence of the ShiLan domain insertion and assessing all six possible reading frames (despite the in-frame translation with the DNA methylase producing no internal stop codons, and hence why this was not performed in our initial investigation) reveals an out-of-frame HNH endonuclease encoded starting 18 bp into the sequence (positions 18, 19, and 20 being GTG for all, except phage Mantra), making it in the third frame relative to the methylase. The last four nucleotides of the insertion sequence are TAAC in all but Mantra and Starchevich, with TAA serving as the stop codon for the HNH endonuclease, yielding a 195 aa product.

We performed several identical database searches using the original frame and HNH frame protein sequences as separate queries to compare the results (BLASTP against NCBI’s non-redundant protein databank [20], Foldseek [21], HHpred [22], and NCBI conserved domain search [27]) (Table 1). Many of these searches were performed in our initial work on the ShiLan domain in its original frame and were repeated here to have updated results to compare directly to those generated using the HNH frame. A multiple sequence alignment of the ShiLan domain HNH endonuclease sequence and hits from the BLASTP search annotated as HNH endonucleases with 70% or greater query coverage is available at the repository for this work [37].

### 3.4. Comparing the Predicted Protein Structure of the Original and the HNH Frame Insertion to Solved HNH Endonuclease Structures

**Figure 3:**
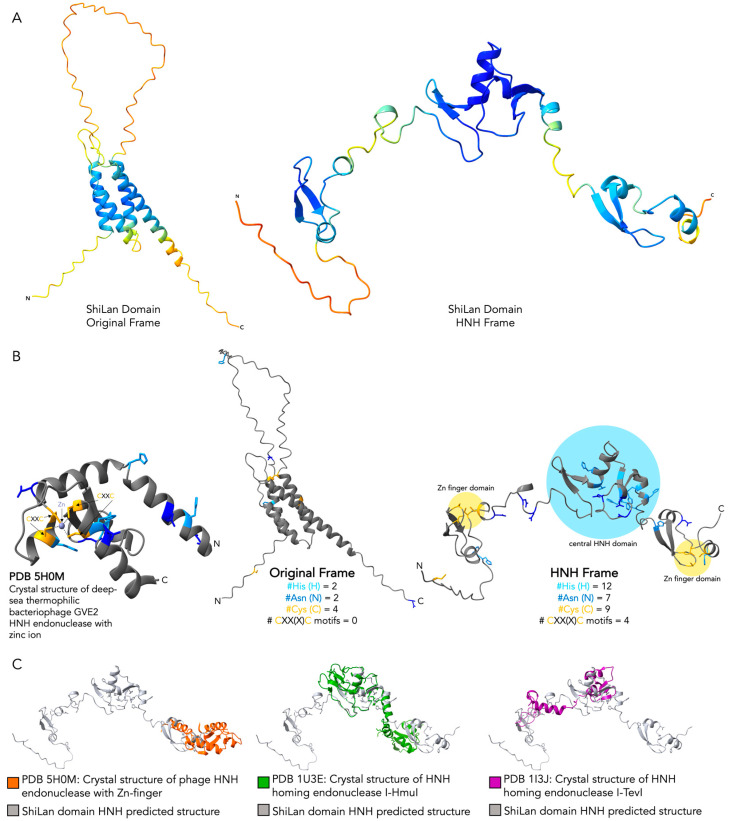
Comparison of the predicted protein structure for the ShiLan domain read in the frame of the methylase and in the alternate frame to solved HNH endonuclease structures. AlphaFold was used to generate predicted protein structures for the ShiLan domain read in the frame of the methylase it invades (original frame) and in the alternate frame encoding an HNH endonuclease (HNH frame). (**A**). The predicted protein structures for the two frames, both colored according to pLDDT model confidence [11]. (**B**). A solved crystal structure of a phage HNH endonuclease with one zinc finger domain, PDB entry 5H0M [16], (left) compared to the structures for the two frames (right). All three structures are colored by residue, with histidine in light blue, asparagine in dark blue, cysteine in yellow, and all others in gray. These residues are important for HNH endonuclease activity, with histidine and asparagine residues comprising the HNH domain, and with pairs of CXX(X)C motifs comprising the zinc finger domains when present. (**C**). The matchmaker tool in ChimeraX was used to align three separate experimentally solved crystal structures of different HNH endonucleases (a phage HNH endonuclease (PDB 5H0M [16]) and two HNH homing endonucleases (PDB 1U3E [17] and PDB 1I3J [18]).

With the database searches providing clear support for the product of the secondary ORF of the insertion having a central HNH endonuclease domain with flanking zinc finger domains, we generated predicted protein structures for the original and HNH frame insertions with AlphaFold [11] (Figure 3A) and used the structural analysis software ChimeraX [13] to compare both structures to the solved crystal structure of a phage HNH endonuclease, PDB entry 5H0M [16] (Figure 3B). The solved HNH structure provides an example of a compact HNH core with a single zinc finger motif. The predicted structure of the product of the ShiLan domain read in the frame of the methylase produced no structural regions resembling either the HNH domain or the zinc finger domain. In contrast, the predicted structure of the product of the ShiLan domain read in the alternate reading frame contains a central HNH domain and two zinc finger domains, all joined by loose linker regions (Figure 3B). This structure also bears strong similarity to the solved structure of larger HNH endonucleases, I-HmuI and I-TevI, which are specifically homing HNH endonucleases (Figure 3C).

### 3.5. Revisiting Possible Intron-Based Scenarios in Light of the HNH Discovery

Group I self-splicing introns are known to commonly contain ORFs encoding homing endonucleases, including HNH endonucleases [39]. These introns are present in some phages including mycobacteriophages [12,40] making their ability to invade this DNA methylase gene and carry this HNH endonuclease ORF feasible. However, we were unable to find computation-based support for these out-of-frame HNH endonucleases being carried by any known type of intron. Group I introns in particular are known to vary in their sequences despite their structural conservation [41] even when invading the same exact insertion site in the same exact gene. As a result, only comparing the nucleotide sequence of an unknown insertion to those of known introns is insufficient. Supplementing sequence comparisons with the comparison of predicted RNA secondary structural elements to those of known introns is important for gathering support for or against the identity of an unknown insertion sequence being a group I intron. As such, we performed sequence-based and RNA predicted secondary structure-based analyses of the ShiLan domain insertion to assess the likelihood of it being an intron.

**Figure 4:**
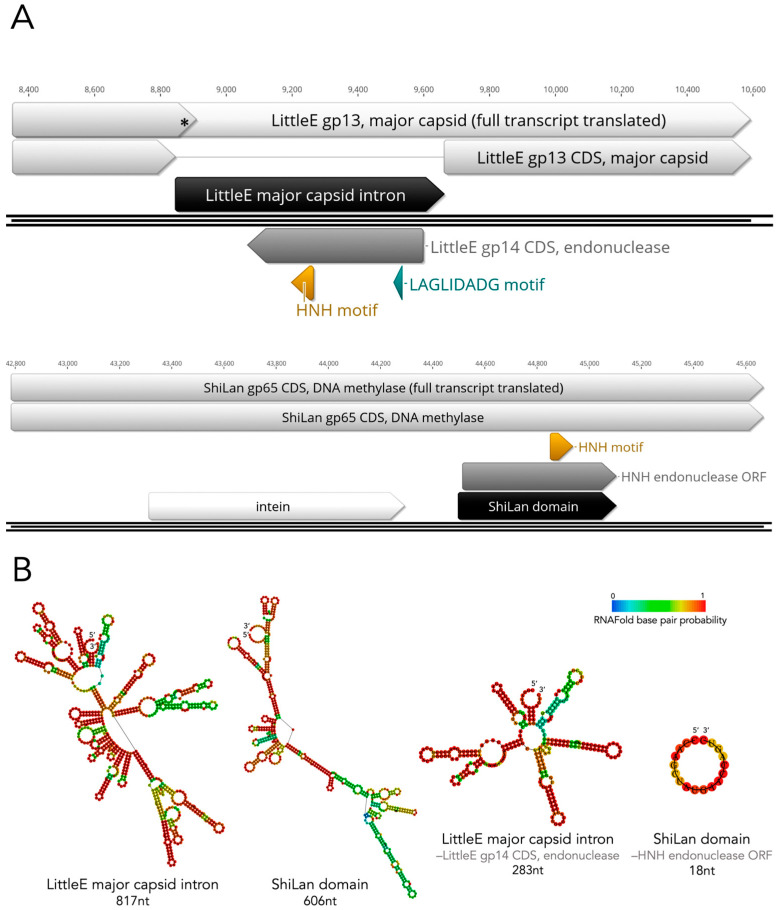
Comparing the predicted RNA secondary structures of the ShiLan domain and a phage group I intron. (**A**). Result of trying to translate through the insertion in LittleE’s major capsid gene gp13 (top) versus translating through the insertions in the ShiLan DNA methylase gene gp65 (bottom). The asterisk indicates where an internal stop codon is reached when trying to translate through the LittleE major capsid gp13 intron. No internal stop codons occur when translating through the ShiLan DNA methylase insertions. (**B**). Minimum-free-energy (MFE)-predicted RNA secondary structures for LittleE’s major capsid gp13 group I intron and the ShiLan domain with (left) and without (right) their ORFs.

The first indication of the ShiLan domain being non-intronic was the fact that methylases containing the ShiLan domain insertion can be translated perfectly from start to finish, i.e., the insertion adds no internal stop codon. In most cases, if one were to try to translate an intronic sequence right in line with its host gene, one would not end up with a seamless protein product. For instance, when the cluster J phage LittleE’s major capsid gene with its group I intron located at nucleotide position 496 is translated from start to finish without removing the intron, a stop codon is reached 30 bp into the insertion, which would truncate the host protein and likely yield a nonfunctional major capsid protein (Figure 4A). In contrast, the seamless translation of methylases with a ShiLan domain/out-of-frame HNH insertion raises the possibility of a non-intron-mediated system.

Nevertheless, to further investigate the ShiLan domain’s potential as an intron, we generated predicted RNA secondary structures for the ShiLan domain and a known phage group I intron (from phage LittleE’s major capsid gene gp13) (Figure 4B). To more clearly visualize the presence and absence of known group I intronic RNA secondary structures, the region of the sequence containing the ORF in each insertion was removed, and a new structure was predicted (Figure 4B). For the ShiLan domain sequence, no intron domains were detected by RNAweasel, and the predicted RNA secondary structure does not closely resemble that of a group I self-splicing intron (Figure 4B) [32,36,41]. In contrast, the LittleE intron was detected as a group I intron by RNAweasel (output available in GitHub repository provided [37]), and the predicted RNA secondary structure bears great resemblance to known group I intron structures.

### 3.6. The Divergent DNA Methylase in Cluster F1 Phage Pacc40 Contains a Different Insertion with a Hidden HNH Endonuclease

**Figure 5:**
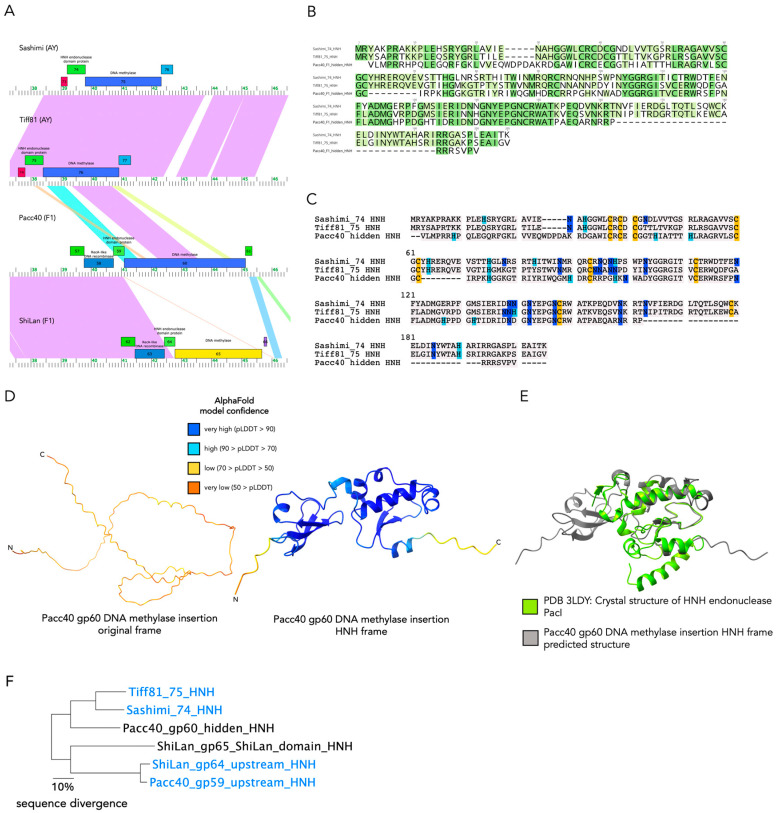
The divergent methylase from cluster F1 phage Pacc40 contains an HNH insertion similar to freestanding HNH endonucleases upstream of the related cluster AY methylases. (**A**). Gene maps generated with Phamerator [29] comparing the similarity between the DNA methylase-containing regions of Sashimi (cluster AY), Tiff81 (cluster AY), Pacc40 (cluster F1), and ShiLan (cluster F1). The Phamerator gene map was edited to remove annotations upstream and downstream and more clearly visualize the region of interest. The shaded regions between two genomes indicate sequence similarity, ranging from purple, having an e-value of 0.0, to red with an e-value of 1 × 10^−4^ [29] Note that Pacc40 gp60 also contains an intein farther downstream, separate from the insertion sequence being discussed here. (**B**). Alignment of the protein product from the Pacc40 methylase insertion sequence read in the HNH endonuclease-encoding frame to the free-standing HNH endonucleases from Sashimi gp74 and Tiff81 gp75, highlighted by sequence similarity, with darker shading indicating the residue being shared across more of the sequences, and lighter shading indicating a less shared residue. (**C**). The alignment [30] described in B colored to highlight histidine (light blue), asparagine (dark blue), and cysteine (yellow) residues, as they are key residues for the HNH motif and zinc finger motifs respectively. (**D**). AlphaFold-predicted protein structures for the insertion sequence in the Pacc40 gp60 DNA methylase read in its original frame (left) and in the putative HNH endonuclease-encoding alternate frame (right). Both structures are colored according to AlphaFold’s pLDDT model confidence scores [11]. (**E**). AlphaFold-predicted structure for the putative HNH endonuclease aligned using Matchmaker in ChimeraX [13] to the experimentally solved crystal structure of the HNH endonuclease PacI (PDB 3LDY [19]). (**F**). Neighbor-joining clustering based on percent pairwise identity calculated from the aligned sequences in Seaview [45] reveals that only the sequences of Sashimi gp74, Tiff81 gp75, and Pacc40 hidden HNH are sufficiently similar to be aligned with confidence.

A different cluster F1 phage, Pacc40, with a divergent DNA methylase (Pacc40 gp60) was found to also have an out-of-frame HNH endonuclease insertion at a different position. Pacc40 gp60 contains a DNA helicase domain followed by a DNA methylase domain, per BLASTP and HHpred searches, thus making its annotation difficult. Similar to the ShiLan domain, the protein sequence of Pacc40 gp60’s insertion sequence when read in-frame with the methylase cannot be assigned any known function (Table 2) and yields a disordered predicted protein structure with poor confidence (Figure 5D). In contrast, an alternate ORF within the insertion yields a 160 aa long protein sequence which retrieved matches to HNH endonucleases and similar proteins (Table 2) and yields a more ordered and confident structural prediction (Figure 5D). The Pacc40 gp60 DNA methylase is included in a different PhagesDB pham (protein family) than the methylases discussed so far, despite this methylase being in the same genomic context. Efforts to align the Pacc40 gp60 DNA methylase to the ShiLan DNA methylase (ShiLan gp65) with all insertion elements removed from both using clustalo [42], MUSCLE [43], and MAFFT [44] alignment algorithms all produced poor alignments (available at the GitHub repository provided [37]). The Pacc40 gp60 methylase is more closely related to DNA methylases found in the cluster AY phages Sashimi and Tiff81 (Sashimi gp75 and Tiff81 gp76) (Figure 5). Interestingly, the phages Tiff81 and Sashimi both harbor an HNH endonuclease similar to that inside Pacc40’s methylase gene (Tiff81 gp75 and Sashimi gp74) upstream of the methylase gene (Figure 5A). Due to the phages Sashimi and Tiff81 having only draft annotations at the time of writing, support for the annotations of Sashimi gp74 and Tiff81 gp75 as HNH endonucleases and Sashimi gp75 and Tiff gp76 as DNA methylases using BLASTP, HHpred, and NCBI Conserved Domain Search as described for Table 1 are provided at the GitHub repository for this work [37]. Additionally, these gp numbers may change in the final versions of the Tiff81 and Sashimi annotations.

We compared the HNH endonucleases that in Tiff81 and Sashimi are encoded directly upstream of the DNA methylase (Tiff81 gp75 and Sashimi gp74) to the HNH insertion in Pacc40 gp60 (Figure 5). The HNH insertion is not present in the Sashimi and Tiff81 methylases (Figure 5A). The annotated beginnings of all three methylases (Pacc40 gp60, Sashimi gp75, and Tiff81 gp76) aligned well, suggesting that this was not a case of misannotation. The intron detection program RNAweasel [33] was used through MFannot [34] on the HNH endonucleases and surrounding regions of Sashimi and Tiff81, as well as the DNA methylases of Sashimi, Tiff81, and Pacc40. No known group I or group II self-splicing intron domains were detected. We would like to note that annotating Sashimi gp74, Tiff81 gp75, and the ORF inside the Pacc40 gp60 insertion as HNH endonucleases specifically is less certain than for the ShiLan domain. Nevertheless, the Pacc40 gp60 insertion remains similar to the ShiLan domain in that the insertion clearly contains a secondary ORF encoding a product with DNA-binding capacity.

## 4. Discussion

### 4.1. The ShiLan Domain May Not Interfere with DNA Methylase Function

**Figure 6:**
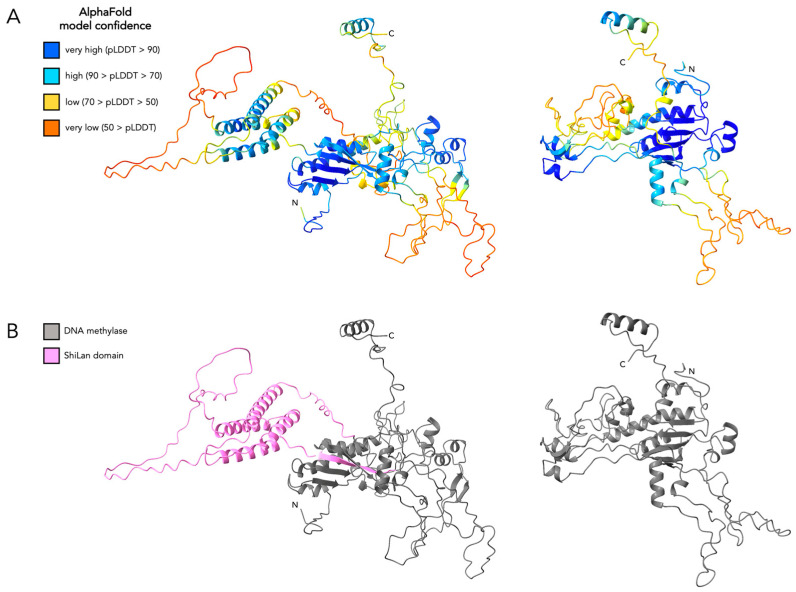
Predicted protein secondary structure of ShiLan gp65 DNA methylase with and without the ShiLan domain insertion. (**A**). AlphaFold was used in ChimeraX to generate a predicted structure for ShiLan gp65 (DNA methylase) with its intein removed and the ShiLan domain included (left) and with its intein and ShiLan domain removed (right). Both structures are colored according to AlphaFold-provided per-residue pLDDT confidence score. (**B**). The structures described in (**A**), with the DNA methylase colored dark gray and the ShiLan domain colored light pink.

As our findings do not suggest the ShiLan domain insertion to be capable of any known post-transcriptional RNA or post-translational protein self-splicing, this suggests that the protein products of ShiLan domain-containing sequences retain the entire ShiLan domain. A key component to the success of an intein is its ability to post-translationally remove itself from its host protein through protein splicing, meaning that it does not hinder the host protein’s ability to function. If the ShiLan domain is not spliced out of the DNA methylase at any step from transcription through post-translation, as we predict to be the case, the ShiLan domain could possibly interfere with DNA methylase activity. To investigate this, we generated predicted protein structures for the ShiLan gp65 DNA methylase (with its intein removed) with and without the ShiLan domain and compared them (Figure 6). These structural predictions suggest that the ShiLan domain may form a hairpin-like structure that is distanced from the rest of the methylase by connecting loops. This distance, which may prevent interference by the ShiLan domain, coupled with this DNA methylase not being essential for phage function (as evidenced by close relatives of phage ShiLan that lack any homolog to the ShiLan gp65 DNA methylase [8]), may sufficiently minimize any decrease in methylase function and phage fitness, such that individuals with ShiLan domain insertions are able to persist in populations.

### 4.2. DNA Methylases and HNH Endonucleases in Phages

DNA methylases are often part of restriction-modification (RM) systems in bacterial genomes, serving as an important component of bacterial defense systems. These RM systems can be horizontally acquired and utilized by phages, which makes RM systems and their methylases an important component in the evolutionary arms race between bacteria and the phages that infect them [46]. DNA methylases can also exist as orphan methylases not associated with an RM system, commonly seen in phages [47]. While not yet known to strongly associate with methylases, HNH endonucleases are a class of highly specific DNA-binding proteins with a variety of known functions. In the context of phages, known HNH endonuclease functions range from aiding terminases in DNA packaging [16] to driving self-propagation of self-splicing introns as homing endonucleases [39]. While the disjoint distribution of these insertion sequences with hidden HNH endonucleases encoded in alternate frames suggests that they have not yet become a fixed element vital to the functioning of the host protein or phage, one could imagine the hidden HNH endonuclease as yet another DNA-binding module to be shuffled around and utilized by methylases and their phages.

### 4.3. How Are Hidden HNH Endonucleases Translated?

A question that arises is that of how these HNH endonucleases are actually translated. Their distribution and evolutionary history indicate that they are in fact spreading as selfish elements [8], likely by homing, which requires them to be currently or recently viable as translated functioning proteins. However, the version of the insertion element made during the translation of the full host gene and insertion transcript is not an HNH endonuclease, meaning that the transcript must also be able to be read in the HNH endonuclease frame. Some bacteria, including *Mycobacterium*, engage in what is known as leaderless initiation, where the ribosome is recruited to translate some transcripts without any ribosomal binding site (RBS) being detectable at the sequence level (e.g., no identifiable Shine-Dalgarno sequence). It is suspected that local secondary structures in the RNA folding are able to recruit the ribosomal assembly and perform successful translation [48]. Since there is no detectable RBS sequence near the HNH endonuclease, leaderless translation initiation is likely important for the production of these proteins. Interestingly, all the PhagesDB phages covered in this work, which contain a methylase with the ShiLan domain and hidden HNH endonuclease, are reported as being able to infect *Mycobacterium*. In addition, all of these phages are also reported on PhagesDB as temperate, meaning that these phages are capable of making prophages in their host genome. This would increase the potential opportunities for the translation of the HNH endonuclease from the transcript, as could occur during lytic infection and phage replication or from the copy of the HNH-containing gene integrated into the host genome within the prophage region. As shown in Table 1, several of the HNH matches identified using the ShiLan domain HNH reading frame product as query are from bacteria; thus, further investigation may reveal some of these to be in prophages. HNH homing endonucleases, as well as other homing endonucleases, are known for being extremely selfish elements that are incredibly successful at self-propagation when given any opportunity for recombination. In *Mycobacterium smegmatis* specifically (infected by ShiLan and the other discussed phages in which these elements have been found), leaderless translation initiation is more effective with the non-canonical start codon GTG than it is with ATG [49,50], and GTG is the exact codon that we propose to be the starting point for the translation of these out-of-frame HNH endonucleases from the larger transcript. The question of ORF translation is also still one of debate for self-splicing introns such as that of mycobacteriophage LittleE’s major capsid protein [12], to which leaderless initiation is one of the more probable answers. For out-of-frame inserted proteins and intron-encoded proteins, *Mycobacterium* seems to be a particularly accommodating and well-equipped host.

### 4.4. Outlook

Our analyses support future experiments to explore the function of these out-of-frame HNH endonucleases and the unlikely possibility of self-splicing of the insertion sequence. Splicing at the RNA level can be addressed through RT-PCR. The absence of splicing at the RNA and protein levels can be verified through the analysis of the expressed methylase. The HNH-encoding ORF expressed in a heterologous system might be used to explore activity and site specificity of the HNH endonuclease.

## 5. Conclusions

Our analyses show that the selfish mobile genetic insertion element that we discovered in the Shilan methylase is actively homing between divergent phage clusters. Our findings reinforce the observation that overlapping reading frames often observed in viruses [51,52] also occur frequently in Actinobacteriophages [7,53].

## Figures and Tables

**Table 1:** Results of database searches using the amino acid sequence encoded in the ShiLan domain read either in the original frame (frame of the methylase gene) or in the alternate frame that encodes a putative HNH endonuclease. BLASTP with default settings (includes maximum 100 target sequence) was used to search against NCBI’s non-redundant protein sequence database. For Foldseek, default search settings were used, and the results from the search against the protein databank (PDB) specifically are highlighted. HHpred with default settings was used to search against the PDB, UniProt’s Swiss-Prot viral protein databank, PHROGs, and PfamA. NCBI’s conserved domain (NCBI CD) search tool was used to search against NCBI CD. Output from each search is available in the provided repository for this work [37].

Program	ShiLan gp65 (DNA Methylase) ShiLan Domain Insertion Original Frame	ShiLan gp65 (DNA Methylase) ShiLan Domain Insertion HNH Frame
BLASTP	9 hits DNA methylase (2) DNA methyltransferase (5) Methyltransferase (2) e-value: 1 × 10^−129^–2 × 10^−58^ query cover: 99–100% spans: phages	100 hits HNH endonuclease (89) Hypothetical protein (11) e-value: 4 × 10^−51^–1 × 10^−12^ query cover: 34–86% spans: phages, bacteria, archaea
Foldseek (PDB100)	0 hits with e-value < 1	1 hit with e-value < 1 × 10^−4^ HNH homing endonuclease I-HmuI (PDB entry 1U3E [17]) e-value: 9.13 × 10^−3^ spans residues 75–173 of query
HHpred	0 hits with e-value < 1	51 hits with e-value < 1 × 10^−4^ HNH endonuclease (38) His-Me/Zn binding (2) endonuclease (7) uncharacterized protein (4) e-value: 8.8 × 10^−5^–1.4 × 10^−13^
NCBI Conserved Domain Search	0 hits with e-value < 1	1 hit HNHc superfamily e-value 5.05 × 10^−4^ spans residues 93–134 of query

**Table 2:** Results of database searches using the amino acid sequence encoded in the Pacc40 gp60 methylase insertion sequence read either in the original frame (frame of the methylase gene) or in the alternate frame that encodes a putative HNH endonuclease. The methods described for Table 1 were applied in these searches.

Program	Pacc40 gp60 (DNA Methylase) Insertion Sequence Original Frame	Pacc40 gp60 (DNA Methylase) Insertion Sequence HNH Frame
BLASTP	4 hits DNA methyltransferase (4) query cover: 95–100% spans: phages and bacteria	100 hits AP2 domain-containing protein (5) HNH endonuclease (2) homing endonuclease (1) hypothetical protein (92) query cover: 87–97% spans: phages and bacteria
Foldseek (PDB100)	0 hits with e-value < 1	0 hits with e-value < 1 × 10^−4^
HHpred	0 hits with e-value < 1	14 hits with e-value < 1 × 10^−4^ HNH endonuclease (5) restriction endonuclease (1) unknown function (8) e-value: 2.3 × 10^−25^–2.2 × 10^−5^ spans residues 4–151 of query
NCBI Conserved Domain Search	0 hits with e-value < 1	0 hits with e-value < 1

## Data Availability

All sequence data used in this study are available at PhagesDB.org and at the NCBI. Alignments, AlphaFold-predicted structures, discussed annotation validation for phages Sashimi and Tiff81, sequence alignment of the ShiLan domain HNH endonuclease to highly similar HNH endonucleases retrieved in the BLASTP search, and complete output from searches covered in Table 1 and Table 2 are available at https://github.com/daniellearsenault/2024_ShiLan_update (accessed on 22 January 2025) [37].

## Abbreviations

The following abbreviations are used in this manuscript:

aa	amino acid(s)
BLASTP	Basic Local Alignment Search Tool for Protein Sequences
bp	base pair(s)
gp	gene product
HNH	type of HE named after a conserved amino acid sequence motif
LAGLIDADG	type of HE named after a conserved amino acid sequence motif
nt	nucleotide(s)
ORF	open reading frame
PDB	Protein Data Bank
pLDDT	predicted Local Distance Difference Test
RBS	ribosomal binding site
RM	restriction-modification
SEA-PHAGES	Science Education Alliance—Phage Hunters Advancing Genomics and Evolutionary Science
